# Concentration of Tea Extracts by Osmotic Evaporation: Optimisation of Process Parameters and Effect on Antioxidant Activity

**DOI:** 10.3390/membranes7010001

**Published:** 2016-12-28

**Authors:** Marisa P. Marques, Vítor D. Alves, Isabel M. Coelhoso

**Affiliations:** 1 LAQV-REQUIMTE, Departamento de Química, Faculdade de Ciências e Tecnologia, Universidade NOVA de Lisboa, Caparica 2829-516, Portugal; cmpe.marques@campus.fct.unl.pt; 2 LEAF—Linking Landscape, Environment, Agriculture and Food, Instituto Superior de Agronomia, Universidade de Lisboa, Tapada da Ajuda, Lisboa 1349-017, Portugal; vitoralves@isa.ulisboa.pt

**Keywords:** osmotic evaporation, membrane contactors, tea extract concentration, antioxidant activity, phenolic content

## Abstract

In this work, the concentration process of three different tea extracts (medicinal Rosil No. 6, Black, and Forest Fruit teas) using the osmotic evaporation (OE) process, was studied. The effect of the OE process on the content of phenolic compounds and antioxidant activity was evaluated. The concentration process was carried out in a hollow-fibre membrane contactor with an effective surface area of 0.54 m^2^. The tea extract was circulated through the shell side of the contactor, while a concentrated osmotic solution (CaCl_2_ 5 M) was circulated inside the fibres. The flux, the driving force, and the mass transfer coefficient were evaluated. A decrease of the water flux over time was observed and was attributed only to the decrease of the driving force, caused by the dilution of the osmotic solution. Using a surface area/feed volume ratio of 774 m^2^·m^−3^, it is possible to reach a tea concentration of 40% (*w*/*w*) in 5 h, with a constant water flux and without losing the phenolic content and antioxidant potential in most teas.

## 1. Introduction

Tea consumption dates from the 27th century B.C., and today remains in the diet of millions of people, being one of the oldest beverages produced by biotechnological methods [1]. Tea is globally one of the most consumed beverages and its consumption occurs mainly in the form of infusion. The interest in tea comes mainly from its aroma, as well as the beneficial effect that the beverage can have on the health of consumers.

Nearly half of the dry matter of tea is insoluble in water and some of its constituents are polyphenols, amino acids, caffeine, sugars and fatty acids. The polyphenols represent about 25% of dry matter and belong mostly to the group of flavonoids or catechins depending on the type of tea.

Generally, the important antioxidant potential present in most teas results from their content in phenolic compounds, chemical structures critical in the absorption and neutralisation of free radicals.

The world production of tea has increased more than consumption, so there is the need to improve product quality and to find innovative ways of processing and distributing this product. Soluble powder tea has been produced and can be consumed as instant soluble herbal tea, as well as a pharmaceutical product [2]. China is one of the countries with the highest consumption of tea and with the higher research focus in this field. It presents a strong industrial activity in the concentration process of teas, with an annual production of about 15,000 tons of concentrated tea, with a concentration of 60 °Brix [3]. Thus, concentrated teas are a convenient form for the consumption of liquid tea, especially as fresh.

From the commercial point of view, the beverages’ concentration is of great interest due to the decreased transportation, storage, and packaging costs, as well as the increase in the product’s shelf life, owing to its higher resistance to microbial activity [4,5,6].

During the beverages’ concentration process, water must be selectively removed, thus making it possible for the consumer, through water addition, obtain a beverage similar to the original, in terms of appearance, flavour, and nutritional quality [7]. At an industrial level, tea aqueous extract initially passes through an aroma-stripping step, followed by concentration with thermal evaporation at low pressure. For the production of powdered tea, the concentrated is dried by spray drying [2]. However, it is known that thermal treatments lead to a considerable loss of heat-sensitive bioactive compounds, which are present in tea.

The industrial processes used currently to concentrate beverages typically involve multistage vacuum evaporation [8]. This commercial concentration process is based on a heat treatment, thereby implying alterations of the quality of the concentrated beverages, both in terms of organoleptic properties, such as colour and aroma compounds, and phenolic compounds content [9,10]. Moreover, these industrial processes involve high energetic costs that emphasise their disadvantages [5].

Membrane processes that have appeared over time enable improvements to the quality of the beverage, improving aspects like colour, flavour, and aroma, maintaining the quality of the processed beverage more close to that of the original. Thus, membrane processes should be considered promising technologies, and, in particular, the osmotic evaporation process emerges as a relevant method because of its advantages when compared to other membrane processes. The osmotic evaporation process allows the removal of water from aqueous solutions by a driving force generated under ambient conditions of pressure and temperature. This allows obtaining concentrated beverages without thermal and mechanical damage, while preserving the aroma and nutritional value [2,6,11,12].

The process of osmotic evaporation has already been used for the concentration of liquid food, especially fruit juices, but also teas, milk, and coffee, either alone [6,7,13,14,15] or integrated with different processes [5,16,17].

The aim of the present work is to study the potential of the osmotic evaporation process for the concentration of teas, assessing its effect on the overall quality of the concentrate obtained. The work has three specific objectives: (i) to evaluate the optimal hydrodynamic conditions; (ii) to study the concentration process of three different teas (Medicinal Rosil No. 6, Black tea, and Forest Fruit tea) by osmotic evaporation, using the optimised operating conditions; (iii) to evaluate the bioactive compound content and antioxidant activity throughout the concentration process; and (iv) to design the operation conditions and scale up to perform a process that concentrates the product up to 40% (*w*/*w*).

## 2. Materials and Methods

### 2.1. Modelling Water Flux and Mass Transfer Resistance

The process of osmotic evaporation uses a porous hydrophobic membrane that separates the solution to be concentrated from an osmotic solution, usually a salt solution, with low water activity. Because of the hydrophobic characteristics of the membrane, and taking into account that the water’s surface tension is higher than the surface tension of the material constituting the membrane, aqueous solutions cannot enter the pores, forming a liquid–vapour interface in this process [18]. The water activity difference between the two solutions induces a driving force in the water flux, which is the water vapour pressure difference. At this point, the water evaporates in the solution to be concentrated and the vapour is transported through the membrane, before being condensed in the osmotic solution, aiming for the chemical potential equilibrium. The water flux is proportional to the water vapour pressure difference between both phases:

(1) $$J_{w}=K_{p}\left(P_{1}-P_{2}\right)$$

where *K_p_* is the overall mass transfer coefficient (m·s^−1^·Pa^−1^) and *P*_1_ and *P*_2_ correspond, respectively, to the water vapour pressure of the solutions in the shell side and in the fibres (Pa). Concerning the water vapour pressure, it can be related to the water activity according to Equation (2):

(2) $$P_{i}=\;a_{i}P_{i}^{*}$$

where *a_i_* is the water activity in the two phases and P_i_* is the pure water vapour pressure (Pa), the latter given by the respective Antoine equation (Equation (3)) [19]:

(3) $$P^{*}=exp[23.2-\left(\frac{3816.2}{T\left(K\right)-46.1}\right)]$$

Due to the concentration polarisation phenomena, the water activity at the membrane interface is different from the bulk water activity resulting on two diffusional boundary layers. The water flux may be calculated using the respective individual mass transfer coefficients:

(4) $$J_{w}=\;k_{s}\left(a_{s}-a_{ms}\right)=k_{t}\left(a_{mt}-a_{t}\right)$$

where *k_s_* and *k_t_* are the individual mass transfer coefficients in the boundary layers (m·s^−1^).

The effect of concentration polarisation phenomenon is closely related to the operating conditions. In order to minimise the concentration polarisation phenomenon, the Reynolds number (Re) on both sides of the membrane should be increased. Combining Equations (1) and (4), the overall mass transfer coefficient can be obtained as:

(5) $$K_{p}=\frac{1}{\left(\frac{P_{ws}^{*}}{k_{s}}\right)+\left(\frac{1}{k_{mp}}\right)+\left(\frac{P_{wt}^{*}}{k_{t}}\right)}$$

where *K_p_* is the overall mass transfer coefficient (m·s^−1^·Pa^−1^); P*_ws_ and P*_wt_ are the pure water vapour pressures at both sides of the membrane (Pa); and *k_mp_* is the membrane mass transfer coefficient (m·s^−1^·Pa^−1^) that may be estimated by [20]:

(6) $$k_{mp}=\frac{1.8\times 10^{-5}}{RT\delta }{\left[\frac{3\tau }{\varepsilon d_{p}{\left(\frac{8RT}{\pi M_{w}}\right)}^{1/2}}+\frac{\tau \rho_{air}}{\varepsilon \;P\;D_{w-air}}\right]}^{-1}$$

where *d_p_* is the pore size (m); ε is the porosity; *p_air_* is the air partial pressure (Pa); *τ* is the tortuosity; *δ* is the membrane thickness (m); *D_w-air_* the water vapour diffusion coefficient in air; *M_w_* is the water molar weight (kg·mol^−1^); *R* is the gas constant (JK^−1^·mol^−1^); *T* the temperature (K); and *P* is the total pressure (Pa).

Considering a situation where the concentration polarisation is negligible (which means that resistance occurs only in the membrane), the overall mass transfer coefficient is equivalent to the membrane mass transfer coefficient. If the boundary layers cannot be neglected, the dependence of the individual mass transfer coefficient of both sides of the membrane can be correlated according to the general mass transfer correlation:

(7) $$Sh=\alpha Re^{\beta }Sc^{\gamma }{\left(d/L\right)}^{\gamma }$$

where *Sh* is the Sherwood number, *Re* the Reynolds number and *Sc* is the Schmidt number, and *α*, *β*, and *γ* are constants.

### 2.2. Osmotic Evaporation Set-Up and Experimental Procedure

**Figure 1:**
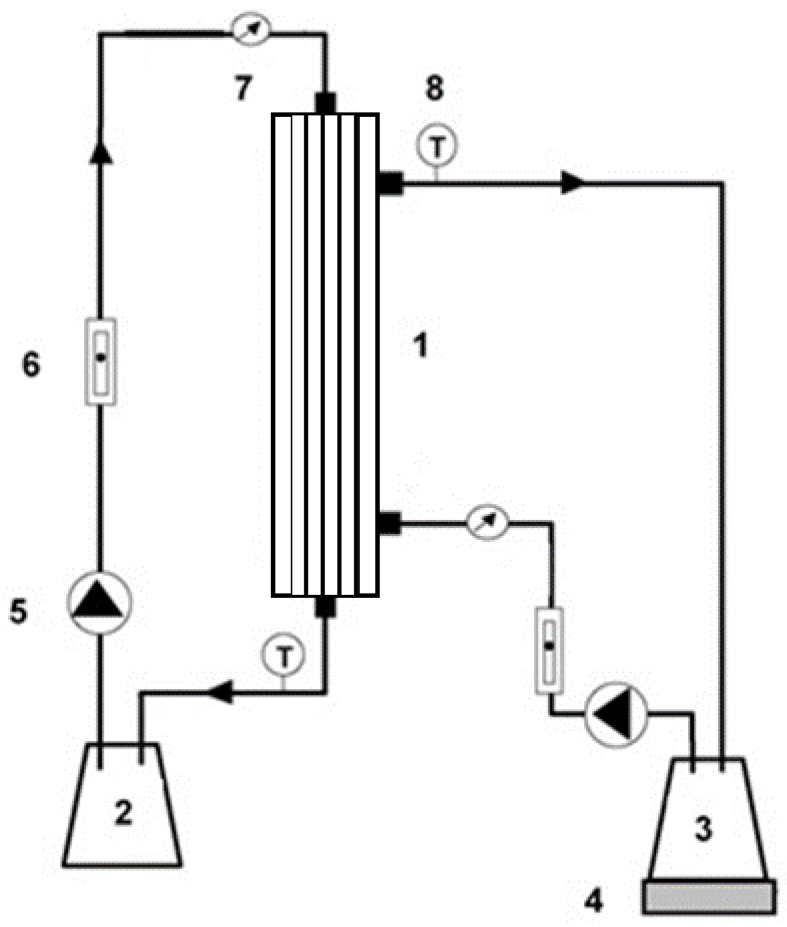
Experimental setup: (**1**) membrane contactor; (**2**) calcium chloride solution reservoir; (**3**) water, sucrose, or tea solution reservoir; (**4**) balance; (**5**) pump; (**6**) flow meter; (**7**) pressure gauge; (**8**) thermocouple.

The experimental setup for osmotic evaporation process consists on a hollow fibre membrane contactor (1.7 × 55 MiniModule, 3M Deutschland GmbH, Wuppertal, Germany) with an effective surface area of 0.54 m^2^ (Figure 1). This membrane contactor contains 7400 polypropylene fibres (Celgard X50-215) with a nominal pore size of 0.04 μm, 40% porosity, 18 cm in length, an internal diameter of 220 μm, and a thickness of 40 μm.

The solutions were prepared with anhydrous calcium chloride (Panreac, Barcelona, Spain) (concentrations 2.5–6.7 M), deionised water, and sucrose (concentrations of 10% (*w*/*w*), 20% (*w*/*w*), and 45% (*w*/*w*)). They were pumped through the shell side of the module (water, sucrose solution, or teas) and lumen of the fibres (calcium chloride solution) in a counter-current mode.

In the optimisation phase, the initial volume of the osmotic solution was 4 L in each experimental run. Inlet and outlet pressures of both tube and shell sides, as well as temperature (23 °C ± 2 °C), were measured during the experimental runs. In order to evaluate the water flux, the weight loss of the feed solution was acquired over time with a balance (Kern, Balingen, Germany) placed under the feed solution vessel. After each experiment, the laboratory setup was cleaned by rinsing both sides with deionised water. The conductivity of effluent water was measured and the cleaning process was finished only when the conductivity was equal to the value of deionised water entering the module. In a first stage, the water flux was measured, keeping constant the Reynolds number on the shell side (Re_shell_ = 2.16), on which flows deionised water, and varying the Reynolds number on the tube side (0.2 < Re < 2.8), for different calcium chloride solution concentrations. In a second stage of the experimental work, the Reynolds number on the fibres side was kept constant (Re_fibres_ = 0.9) and the water flux was measured varying the Reynolds number on the shell side (0.1 < Re < 3.8) where sucrose solutions with different concentrations were circulated.

During tea concentration process, the tea extracts were recirculated in the shell side of the membrane module and a 5 M calcium chloride solution (Panreac, Barcelona, Spain) recirculated in the lumen of the fibres (Re_fibres_ = 0.9 and Re_shell_ = 2.6). The initial volume of the osmotic solution was 8 L in each experimental run and the initial tea volume was 2 L for each tea. After each experiment, the laboratory setup was cleaned by rinsing both sides with deionised water. The conductivity of effluent water was measured and the cleaning process was finished only when the conductivity is equal to value of deionised water entering the module.

The concentration of all solutions was measured with a refractometer (Pal-α pocket, Atago, Tokyo, Japan) and the water activity was measured with a water activity meter (Hygropalm aw, Rotronic, Bassersdorf, Germany).

### 2.3. Extraction Process of Teas

Commercial Black tea (tea bags, Lipton, Uniliver Portugal, Lisbon, Portugal), Forest Fruit tea (tea bags, Lipton, Uniliver Portugal, Lisbon, Portugal), and Medicinal Rosil No. 6 tea (dried leaves, herbal Rosil) were used. Their main ingredients are presented in Table 1.

Regarding Black and Forest Fruit teas, the bags taken from the market were opened and the content was weighed (1.64–2.02 g per bag). The content of one bag was added to 200 mL of water; and the infusion time was 2.5 min in boiling water. The obtained extract was cooled to room temperature. The total soluble solids content (TS) of the extracts were 0.3 °Brix and 0.2 °Brix, respectively. The Medicinal Rosil No. 6 tea extract was obtained by adding 8.15 g of dry leaves to 1000 mL of water and boiling for 5 min, after which the mixture was allowed to infuse for 2 min. The obtained extract was cooled to room temperature and subsequently filtered with filter paper. The concentration of the soluble solids was 0.3 °Brix, as determined using a refractometer (Pal-α pocket, Atago, Tokyo, Japan).

### 2.4. Total Phenolic Content

The total phenolic content was determined using the Folin–Ciocalteau method, in which the polyphenols react with the Folin reagent (Panreac, Barcelona, Spain) forming a blue dye [21]. Firstly, a 0.25 M solution of Folin–Ciocalteau reagent was prepared. Then, a volume of 150 µL of this reagent was added to 150 µL of sample and 2.4 mL of nanopure water. The test tubes containing this mixture were stirred with a vortex; the reaction took place for 3 min, after which 300 µL of 1 M Na_2_CO_3_ (Panreac, Barcelona, Spain) was added to each tube. Then, the test tubes were shaken and allowed to react for 2 h in the dark, after which the absorbance was measured using a spectrophotometer (Thermo Fisher Scientific, Waltham, MA, USA) at a wavelength of 725 nm. A calibration curve was prepared using gallic acid as a standard. The results were expressed as gallic acid equivalents (GAE) per mass of total soluble solids present in each sample (g GAE/g TS).

### 2.5. Antioxidant Activity

The Ferric reducing ability power (FRAP) method was performed according to the method of Benzie et al. (1996) [22]; however, some changes were introduced. The following stock solutions were prepared: (i) acetate buffer (Riedel-de Haen, Seelze, Germany), pH 3.6; (ii) 10 mM tripyridyltriazine (TPTZ) solution (Sigma-Aldrich, St. Louis, MO, USA); (iii) 20 mM ferric chloride aqueous solution (Panreac, Spain); and (iv) 40 mM hydrochloric acid (HCl) (Carlo Erba Reagents, Peypin, France). The FRAP reagent solution was obtained by mixing 25 mL of acetate buffer (pH 3.6), 2.5 mL of 10 mM TPTZ solution, and 2.5 mL of 20 mM ferric chloride aqueous solution. This solution was prepared immediately before being used. A volume of 2.7 mL of the FRAP reagent solution was mixed with 270 μL of nanopure water, to which an aliquot of 90 µL of each of the samples was added. The mixtures were placed in the dark for 30 min in a water bath at T = 37 °C. After reaction, the absorbance was measured at 595 nm using a spectrophotometer (Unicam UV/Vis). A calibration curve was performed using ferrous sulphate as reference. The results were reported to the mass of total solids present in each sample and expressed in g ferrous sulphate/g TS.

The DPPH (2,2-diphenyl-1-picryl-hidrazil) method was performed with reference to the method of Brand-Williams et al. (1995) [23]. A stock solution was prepared by dissolving 24 mg of DPPH (Sigma-Aldrich, St. Louis, MO, USA) in 100 mL of methanol (Sigma-Aldrich, St. Louis, MO, USA), which was stored at −20 °C for at least 2 h. The working solution, required for application of the method, was prepared by dilution of the stock solution in methanol at a ratio of (10:45). An aliquot of 150 µL of each tea sample was added to 4 mL of the working solution and stored in the dark for 40 min, so that the antioxidants present in the sample would react with the radical. A triplicate was carried out for each tea. After this time, the absorbance of all samples was measured at 517 nm. A calibration curve was performed using Trolox as reference antioxidant. The results were expressed as Trolox equivalent antioxidant activity (TEAC) and reported to the mass of total soluble solids present in each sample, µM TE/µg TS.

## 3. Results and Discussion

### 3.1. Hydrodynamic Conditions in the Fibres and Shell Sides

For the concentration of aqueous solutions by the osmotic evaporation process, it is imperative to firstly study the effect of the hydrodynamic conditions in the hollow-fibre membrane contactor, in order to select those that minimise the boundary layers’ resistance.

**Figure 2:**
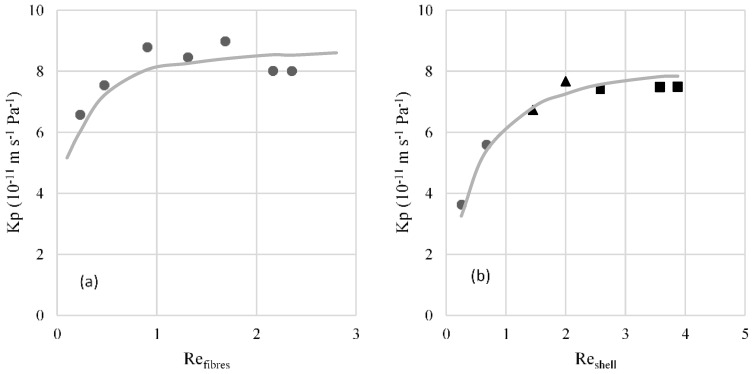
(**a**) Overall mass transfer coefficient (*K_p_*) as a function of Reynolds number (Re) in the fibres. Symbols represent the experimental data and line the data estimated by the correlation $Sh=0.6Re^{1.0}Sc^{0.33}{\left(d/L\right)}^{0.33}$; (**b**) overall mass transfer coefficient (*K_p_*) as a function of Reynolds number in the shell. Symbols represent the experimental data obtained with different sucrose solutions circulating in the shell: ● 45% (*w*/*w*), ▲ 20% (*w*/*w*) ■ 10% (*w*/*w*). The line is the estimated data by the correlation $Sh=0.7{\left(Re\;dh/L\right)}^{0.93}Sc^{0.33}$.

In the study of the hydrodynamic conditions in the fibres, the overall mass transfer coefficient was calculated using Equation (5) and P*_wt_ and P*_ws_ using the average temperatures of the bulk (calcium chloride solution and water, respectively). It was assumed a membrane tortuosity value of 6.5, estimated as τ = (2 − ε)2/ε. The results presented in Figure 2a show an increase of the *K_p_* with the increase of the Reynolds number until it reaches a plateau. The value of this plateau is very similar to the estimated value of the membrane mass transfer coefficient using Equation (6) (8.8 × 10^−11^ m·s^−1^·Pa^−1^), meaning that the mass transfer resistance in both boundary layers may be considered negligible. It may concluded that for Reynolds numbers above 0.9, the mass transfer resistance on the fibres side may be considered negligible.

For the study of the more favourable hydrodynamic conditions on the shell side, the Reynolds number was fixed at 0.9 for the calcium chloride solution that circulates inside the fibres. The values of *K_p_* obtained are represented in Figure 2b. The overall mass transfer coefficient shows a higher dependence with the Reynolds number due to the higher mass transfer resistance on the contactor’s shell side. The obtained *K_p_* value for the reached plateau is similar to the value of the estimated membrane’s mass transfer coefficient (8.8 × 10^−11^ m·s^−1^·Pa^−1^), with a deviation of 14%. In addition, it can be observed that the plateau is reached for Reynolds numbers above 2, for which mass transfer resistance on the shell side may be considered negligible under the operating conditions studied.

### 3.2. Mass Transfer Correlations

The general correlation represented by Equation (7) was fitted to the experimental data assuming γ = 0.33. The values of water diffusion coefficients in calcium chloride (4.1 × 10^−10^ m^2^·s^−1^ ≤ D_w_ ≤ 1.3 × 10^−9^ m^2^·s^−1^) and sucrose solutions (7.1 × 10^−10^ m^2^·s^−1^ ≤ D_w_ ≤ 2.2 × 10^−9^ m^2^·s^−1^) used were obtained from the literature [24,25].

The correlation that best describes the mass transfer coefficient in the fibres is $Sh=0.6Re^{1.0}Sc^{0.33}{\left(d/L\right)}^{0.33}$ (Figure 2a), which is similar to that described by Viegas et al. (1998) [26] ($Sh=0.2Re^{1.01}Sc^{0.33}{\left(d/L\right)}^{0.33}$). It should be noted that the latter correlation shows a deviation from classical laminar flow, probably due to the nonuniform distribution of the fibres and its possible deformation [26]. This correlation was obtained for very low Reynolds numbers, which also happens in the present work.

The mass transfer correlation for the shell side that describes the experimental results with higher accuracy is $Sh=0.7{\left(Re\;dh/L\right)}^{0.93}Sc^{0.33}$ (Figure 2b). This correlation is quite similar to that obtained by Yang and Cussler ($Sh=1.25{\left(Re\;dh/L\right)}^{0.93}Sc^{0.33}$), which is applied to a high range of Reynolds numbers, including that of this work.

### 3.3. Concentration Process by Osmotic Evaporation

#### 3.3.1. Water Flux, Overall Mass Transfer Coefficient, and Driving Force

**Figure 3:**
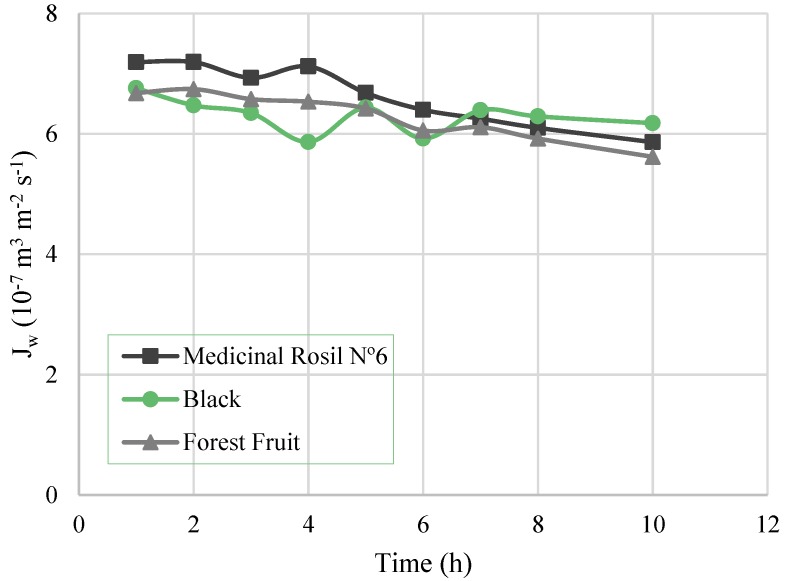
Water flux as a function of the time.

The evaluation of the osmotic evaporation process itself involves the analysis of the water flux, driving force, and overall mass transfer coefficient. Figure 3 shows the permeate flux over time, for the three experiments. A flux decrease after 4 h of operation for Medicinal Rosil (from 7.12 × 10^−7^ m^3^/m^2^·s at 4 h to 5.86 × 10^−7^ m^3^/m^2^·s at 10 h) and Forest Fruit (which decreased from 6.53 × 10^−7^ m^3^/m^2^·s at 4 h to 5.62 × 10^−7^ m^3^/m^2^·s at 10 h) was noticed. Overall, in 10 h of operation, the concentration process of Medicinal Rosil No. 6 tea registered a flux decrease of about 22%, which was 16% and 8.8% for Forest Fruit and Black tea, respectively.

**Figure 4:**
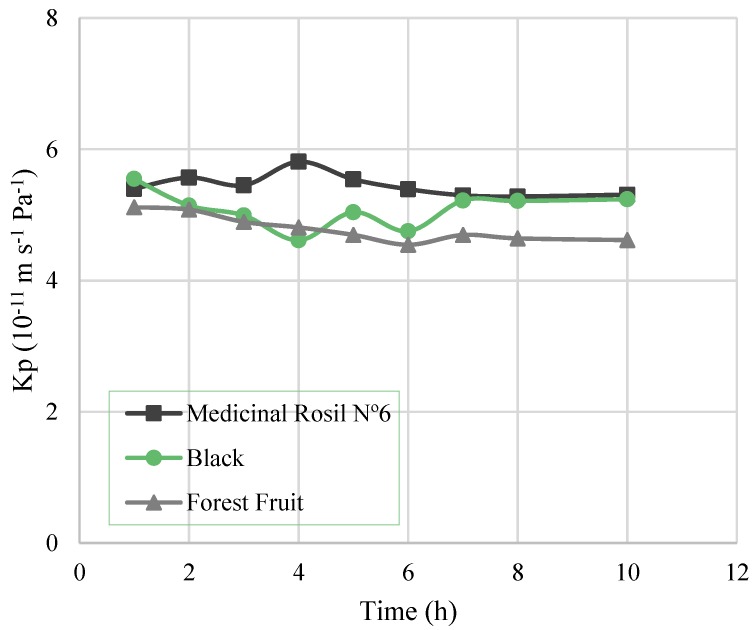
Overall mass transfer coefficient as a function of the time.

Regarding the overall mass transfer coefficient, *K_p_* (Figure 4), it is possible to observe that for all the experiments it remained practically constant throughout the experiment. Thus, the decrease of the water flux is not due to the increase of mass transfer limitations over time. Still, the value of the overall mass transfer coefficient for all the experiments (5.37 ± 0.23, 5.14 ± 0.23, and 4.75 ± 0.25 (10^−11^ m^−1^·s^−1^·Pa^−1^) for Medicinal Rosil No. 6, Black, and Forest Fruit teas, respectively) was lower than the estimated mass transfer coefficient of the membrane (8.88 × 10^−11^ m^−1^·s^−1^·Pa^−1^). This fact indicates the existence of mass transfer resistance in the shell side boundary layer adjoining the membrane, where tea is circulating, right from the beginning. This is possibly due to fouling caused by the deposition on the membrane of molecules and components in suspension in the tea extracts.

**Figure 5:**
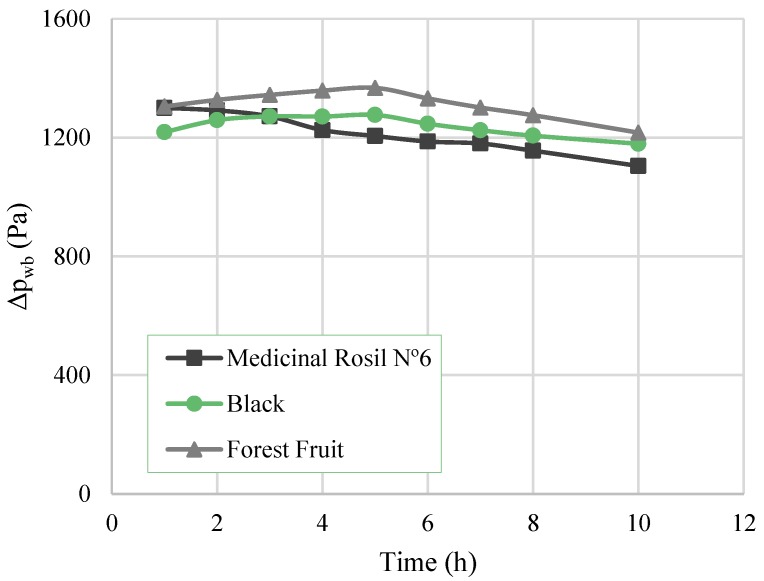
Overall driving force as a function of the time.

The driving force value over time for each concentration process is presented in Figure 5. A decrease tendency can be observed, which is in line with the evolution of the water flux. The decline of the flux is mainly attributed to the dilution of osmotic agent and to the concentration of tea extract over time, similar to the results obtained by Cassano et al. (2010) [11] and Torun et al. (2014) [2]. The dilution of the osmotic agent led to the decrease of the water vapour pressure of the osmotic solution, which resulted in the decrease of driving force for water transport.

Throughout the concentration process, there was an increase of the total soluble solids (TS) of the teas. Comparing the values, based on 10 h of operation, there was an increase of 3.6 times from the initial TS for the Medicinal Rosil No. 6 tea, while Black and Forest Fruit teas had an increase of 3.4 and 3.2 times respectively.

#### 3.3.2. Total Phenolic Content

**Figure 6:**
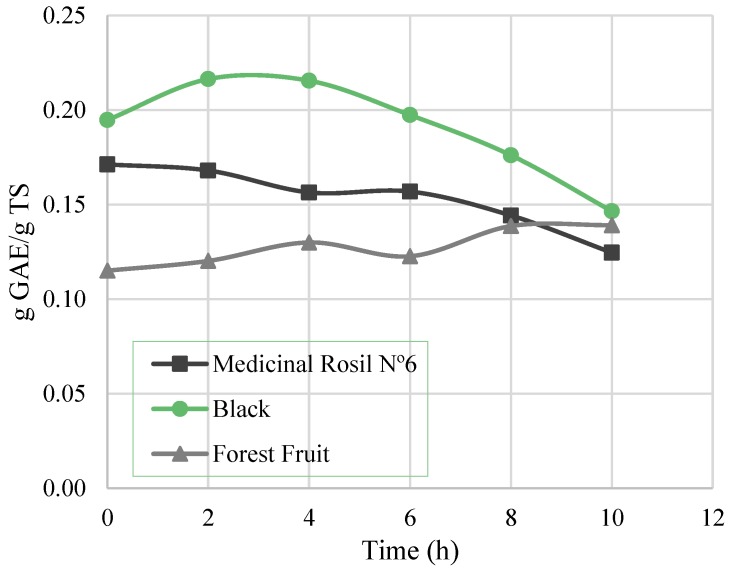
Total phenolic content through the concentration process.

Figure 6 shows the variation of total phenolic content over time for the three concentration processes. A decrease of total phenolic content can be observed for Medicinal Rosil No. 6 and Black teas. For the latter, the decline was observed only after 6 h of processing, decreasing thereafter until the end of the experiment, registering a 24.8% loss in phenols. For the Medicinal Rosil No. 6 tea, in the first 6 h, only 8.4% of total phenols was lost, while for the test time between 6 and 10 h a greater loss of phenols is observed, resulting in a total loss of 27.3%. The decrease in the phenolic content for long processing times (more than 6 h) may be attributed to degradation of these compounds. Still, for Forest Fruit tea, there was no substantial decrease of the total phenolics noticed over time.

The results obtained in this work (0.12–0.22 g GAE/g TS) are generally lower than those obtained in other studies (0.2–0.4 g GAE/g TS) [2,27]. These differences are essentially due to the different plants used to produce the teas. In addition, when the same plant material is used, the differences in the phenolic composition may arise from the year and local of plants harvest. From the results of Figure 6, it is important to highlight that if the process was carried out in two hours, a maintenance of the phenolic content higher than 94% could be expected, as obtained by Torun et al. (2014) [2] for tea extracts.

#### 3.3.3. Antioxidant Activity

**Figure 7:**
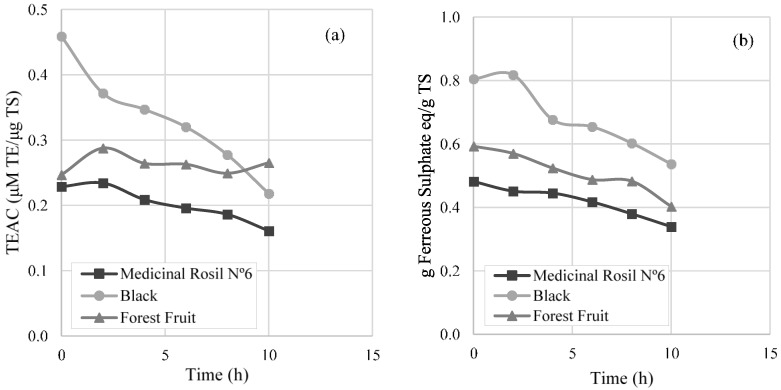
Antioxidant activity through concentration process by (**a**) DPPH (2,2-diphenyl-1-picryl-hidrazil) method and (**b**) Ferric reducing ability power (FRAP) method. TEAC: Trolox equivalent antioxidant activity; TS: total soluble solids.

From the analysis performed by the DPPH test (Figure 7a), a decrease of the antioxidant activity of 29.6% and 52.5% was observed for Medicinal Rosil No. 6 and black teas, respectively, referring to 10 h of operation. On the other hand, the Forest Fruit tea maintained the antioxidant activity during all the concentration process. With regard to the analysis realised by the FRAP method (Figure 7b), a decrease of the antioxidant activity by 29.6% and 33.3% was observed for the Medicinal Rosil No. 6 tea and the Black tea, respectively. However, when analysing the Forest Fruit tea, a decrease of 32.0% of the antioxidant activity is observed, which is not in line with the results obtained by the DPPH method. This fact may be due to the different chemical reactions that occur in each of the methods [27,28,29]. Not all the compounds were able to reduce F3+-TPTZ (ferric-tripyridyltriazine) to the ferrous form (F2+-TPTZ) are antioxidants, and not all the antioxidants that react with the DPPH radical have the ability to reduce the F3+-TPTZ complex [30].

The registered oscillations during the concentration process, referring to the antioxidant activity, might be due to the conversion of antioxidant compounds to others that might have higher or lower antioxidant activity than the group of antioxidant compounds at the initial moment. From Figure 7, it is noticeable that the black tea presents a higher antioxidant capacity, with the Medicinal Rosil No. 6 tea being the one that shows less antioxidant capacity in the initial sample, having only about 50% of the antioxidant activity present in black tea. The Forest Fruit tea was the one in which antioxidant activity remained steady after the concentration process, according to the DPPH method. It seems that the antioxidants that react with DPPH radical present on the Forest Fruit tea are more stable than those present in the other teas.

Factors that may have contribute to the decrease of antioxidant activity in this process at a laboratory scale (e.g., light and oxygen; process time) need to be minimised if the osmotic evaporation process is to be performed at an industrial scale. Taking into account the results obtained, it is evident that during the first 5 h of processing, a low variation of antioxidant activity and total phenols was perceived, so it would be interesting to set up a contactor with a membrane area that enables the production of a concentrated beverage in less than 5 h.

#### 3.3.4. Correlation between Antioxidant Activity and Total Phenolic Content

**Figure 8:**
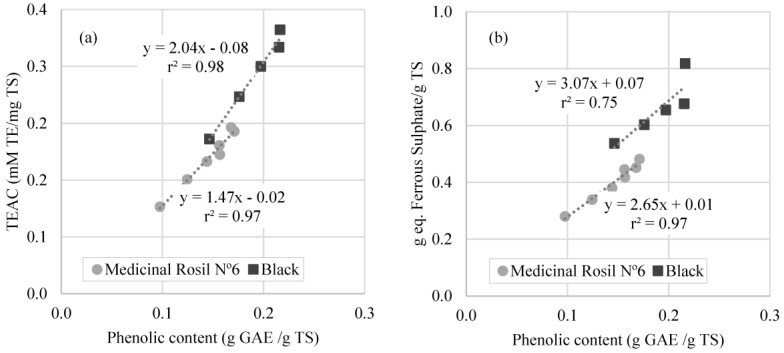
Correlation between phenolic content and antioxidant activity measured by (**a**) DPPH method; (**b**) FRAP method.

According to the literature, there is a strong relation between phenolic content and antioxidant activity, especially in plant extracts [2]. The study of this relation was carried out in this work, using the values obtained for the antioxidant activity as a function of total phenolic content over the concentration processes (Figure 8).

It is possible to verify that the phenolic content determined by Folin-Ciocalteau method correlates with the antioxidant activity measured by the DPPH and FRAP methods for medicinal Rosil No. 6 tea and black tea. Similar results have been reported by Turkmen et al., 2006 [31], which registered a good correlation between the antioxidant activity measured by DPPH method and total phenolic content of black tea. However, this correlation was not obtained in the present work for the forest fruit tea. Similar results to these were reported by Kähkönen et al., 1999 [32] for plant extracts, indicating that different phenolic compounds have different responses in the Folin–Ciocalteau method, and molecular antioxidant response of phenolic compounds varies depending on their chemical structure. This situation leads to the conclusion that, in this work, the phenolic content confers the majority of the antioxidant potential in Medicinal Rosil No. 6 tea and Black tea. On the contrary, for the Forest Fruit tea, the correlation between antioxidant activity and phenolic content is not so evident.

#### 3.3.5. Concentration Process Scale-Up

This experimental work allowed the study of the concentration process of teas regarding several aspects, including hydrodynamic conditions, phenolic content, and antioxidant activity during the concentration process. The study of the hydrodynamic conditions was extremely important for the determination of the operating conditions that enable minimisation of the boundary layers’ resistance and maximisation of water flux values. On the other hand, the antioxidant activity study indicated that the concentration process should not exceed 2 h.

This experimental study for the concentration of teas with the 1.7 × 55 MiniModule has made it now possible to evaluate the required conditions for the tea concentration process up to 40% (*w*/*w*), using the same module specifications and the same hydrodynamic conditions.

To perform an efficient process in the scale-up unit, the driving force should be kept constant by reconcentrating the calcium chloride solution concentration. As such, the water flux can be assumed constant throughout the process. In this way, to achieve a final tea concentration of 40% (*w*/*w*) in 5 h of operation, the ratio between membrane area (A) and initial tea volume (Vi) should be 774 m^2^·m^−3^.

## 4. Conclusions

The concentration of three different tea extracts (Medicinal Rosil No. 6, Black, and Forest Fruit teas) was achieved using an osmotic evaporation (OE) process, carried out in a hollow-fibre membrane contactor with an effective surface area of 0.54 m^2^. The hydrodynamic conditions were previously optimised to the concentration of tea extracts, in order to maximise the mass transfer coefficient. A decrease of the water flux over time was observed, which may be mainly attributed to the decrease of the driving force, caused by the dilution of the osmotic solution.

A decrease in the phenolic content and antioxidant activity for long processing times (more than 5 h) was observed, except for Forest Fruit tea. A surface area/feed volume ratio of 774 m^2^·m^−3^ will be needed to reach a tea concentration of 40% (*w*/*w*) in 5 h, with a constant water flux and without losing the phenolic content and antioxidant potential in most teas.

## Figures and Tables

**Table 1:** Main ingredients of teas.

Tea	Ingredients
Medicinal Rosil No. 6 tea	Avocado (*Persea americana*)
	Boldo (*Peumus boldus*)
	Gorse flower (*Genista tridentata*)
	Horsetail (*Equisetum giganteum*)
	Herb Robert (*Geranium robertianum*)
	St. John’s weed (*Hypericum perforatum*)
	Dandelion (*Taraxacum officinale*)
Black tea	Camellia Sinensis (*Camellia sinensis*)
Forest Fruit tea	Camellia Sinensis (*Camellia sinensis*)
	Raspberry (*Rubus idaeus*)
	Cherry (*Prunus*)
	Blackberry (*Morus*)
	Redcurrant (*Ribes rubrum*)
